# Supplementary material for: Content-rich biological network constructed by mining PubMed abstracts
Source: BMC Bioinformatics. 2004 Oct 8;5:147. doi: 10.1186/1471-2105-5-147 (PMC528731; doi:10.1186/1471-2105-5-147)
Supplement: Additional File 2 — The original results of the above study (non-essential files are deleted to keep the file size under the limit set by BMC bioinformatics). [file 1471-2105-5-147-S2.bz2 › chilibotAdditionalFile2/dip05/169ID8516283E582/html/CDC2_PRKAR2B.html]

 


 **CDC2** and **PRKAR2B** 
  
Found 1 abstracts in PubMed, retrieved 1.  
 

 What does Google say? 
 PDF only 
| .edu only 

---

**Interactive relationship** (e.g. stimulation, inhibition, etc)

**Neutral relationship**- The potential regulatory significance of the phosphorylation of this site on the interaction with microtubule associated protein 2 and other RII anchoring proteins and the physiological relevance of this cyclin B p34cdc2  [ **CDC2** ]  kinase catalyzed modification of RII beta  [ **PRKAR2B** ]  or phosphorylation by other proline directed protein kinases are discussed.  Ref: 8516283 Proc Natl Acad Sci U S A, 1993
- This phosphorylation site matches the consensus sequence X T S PX K R for p34cdc2  [ **CDC2** ]  recognition and belongs to a well conserved sequence found in all RII beta  [ **PRKAR2B** ]  sequences known to date.  Ref: 8516283 Proc Natl Acad Sci U S A, 1993

**Non-interactive relationship** (e.g. studied together, co-existance, homology, etc.)

- By using truncated NH2 terminal RII beta  [ **PRKAR2B** ]  fusion proteins expressed in Escherichia coli and the mitotic protein kinase p34cdc2  [ **CDC2** ]  isolated from HeLa cells or starfish oocytes, we investigated the in vitro phosphorylation of RII beta  [ **PRKAR2B** ]  by these kinases.  Ref: 8516283 Proc Natl Acad Sci U S A, 1993
